# Between-Session Reliability of Common Strength- and Power-Related Measures in Adolescent Athletes

**DOI:** 10.3390/sports5010015

**Published:** 2017-02-14

**Authors:** Christopher Thomas, Thomas Dos’Santos, Paul Comfort, Paul A. Jones

**Affiliations:** Directorate of Sport, Exercise and Physiotherapy, University of Salford, Salford, Greater Manchester M6 6PU, UK; t.dossantos@hotmail.co.uk (T.D.S.); p.comfort@salford.ac.uk (P.C.); p.a.jones@salford.ac.uk (P.A.J.)

**Keywords:** jump height, isokinetics, isometric peak force, countermovement jump, hop tests

## Abstract

The purpose of this study was to investigate the between-session reliability of common strength- and power-related measures in adolescent athletes. Seventeen adolescent athletes (males: *n* = 8: age 17.1 ± 2.2 years; height 175.6 ± 3.5 cm; mass 80.2 ± 3.6 kg; females: *n* = 9; age 16.9 ± 2.6 years; height 178.5 ± 4.3 cm; mass 71.5 ± 4.5 kg) participated in this study. Isokinetic dynamometry, isometric mid-thigh pull (IMTP), countermovement jump (CMJ), and horizontal jumps (standing broad jump (SBJ) and single-leg hop (SLH)) were each performed twice on separate days, seven days apart. Reliability was assessed by intraclass correlation coefficient (ICC), coefficient of variation (%CV), standard error of measurement (SEM), and smallest detectable difference (SDD). Intraclass correlation coefficients and CV demonstrated acceptable between-session reliability for all measures (ICC > 0.63; CV < 11%), except rate of force development and impulse measures during bilateral and unilateral stance IMTP. Smallest detectable differences demonstrated that changes in performance of >7% CMJ height, >8% SLH distance, >10% in peak isometric force, and >5% in isokinetic peak torques should be considered meaningful, when assessing adolescent athletes.

## 1. Introduction

The assessment of physical capabilities such as strength and power plays an important role in the evaluation of training effects. Performance testing can provide a rationale with which to individualize strength training programmes to improve specific physical qualities required for successful sports performance. Frequent assessment of athletes helps strength and conditioning coaches and sport scientists to monitor acute training responses, measure chronic training effects, identify strengths and weaknesses, individualize training programmes, and compare data to normative measures found in peer reviewed publications [1]. Strength and power are key determinants in sporting success [2], but there is a need to accurately assess these qualities in adolescent athletes through valid and reliable testing protocols, such as isokinetic dynamometry, isometric mid-thigh pull, countermovement, and horizontal jump tests. Despite a growing body of evidence in adult populations, these assessments have been used less in male and female adolescent athletes.

Reliability relates to the consistency of the measure, controlled for by the protocol design, clear definition of procedures, equipment calibration, and consistent testing through practice and the use of pilot studies. The standard error of measurement (SEM) provides a direct measure of the amount of error associated with the test, which can also be represented as a percentage of variation in an athlete’s mean score (%CV) [3]. These measurements allow strength and conditioning coaches and sport scientists to make sound conclusions about whether the changes seen in response to a training intervention are meaningful or not [3]. The smallest detectable difference (SDD), refers to the ability of the test to detect the smallest practically important change. When interpreting test results, this value should fall outside the SEM to determine whether changes in testing results are of benefit to athletic performance [4]. Retest correlations such as intraclass correlation coefficients (ICC) refer to the reproducibility of variables for repeated tests, with a value of ≥0.50 being considered highly reliable [5]. However, a test deemed reliable using ICCs does not necessarily suggest it is effective for monitoring performance in athletes. High reliability is not only important for testing procedures, but also when also monitoring changes in individual and team training effects over extended periods of time.

Research has shown isokinetic dynamometry to be a reliable method (ICCs 0.80–0.97) of assessing single-joint muscular strength in a range of populations [6,7,8,9]. Calculation of knee flexor/extensor muscle imbalance appears to be an indicator of increased risk of injury, with an imbalance between the hamstrings and quadriceps (functional eccentric hamstring to concentric quadriceps ratio of <1:1, or a conventional concentric hamstring to concentric quadriceps ratio of <2:3) indicating increased risk of hamstring [10] and anterior cruciate ligament (ACL) injuries [11]. However, the relation of isokinetic strength to dynamic performance still remains unclear due to contrasting biomechanical and neuromuscular characteristics [12]. Sole et al. [9] reported a high reliability (ICC ≥ 0.92) for isokinetic knee flexion and extension between test sessions in 20 healthy subjects, whereas Graham-Smith et al. [13] demonstrated high reliability values (ICC ≥ 0.92; CV ≤ 5.83%; SEM ≤ 14.22 N·m) for isokinetic knee flexor and extensor performance of the right leg in 23 male soccer and rugby athletes. However, a limited number of studies have examined reliability measures of isokinetic dynamometry in adolescent athletes [14,15].

Hop tests are primarily developed to assess horizontal power-related performance, but are also popular within clinical environments to assess and monitor rehabilitation from lower-limb injury. Furthermore, as hop tests are performed unilaterally, this allows quantification of between-limb symmetry, and may help determine patient function and return-to-play decisions when recovering from injury [16]. The single-leg hop (SLH) has been shown to yield a reliable measure of horizontal power-related performance among university students, with a high test-retest reliability (ICC 0.80; SEM < 0.08 m) [11]. Typically, the protocol for the SLH allows use of the arm swing [17], however few studies have specifically assessed SLH performance without the use of arm swing. Research has shown the inclusion of an arm swing during vertical jumps results in increased peak vertical ground reaction force and greater jump heights (~8.6 cm), compared to no arm swing [18]. However, this may be inappropriate when using hop tests to assess unilateral hop performance due to (1) transfer of energy from the arms to the rest of the body during the later stages of the jump, thus increasing potential energy and kinetic energy at the point of take-off; and (2) inclusion of an arm forward inclination of the trunk, enabling it to extend earlier and generate power more quickly and over a longer period of time [19]. Identification of reliability measures is therefore essential to ensure that sport scientists and practitioners apply appropriate methods when collecting baseline data and to establish the measurement error inherent in the testing procedures adopted.

One of the most commonly used tests to measure isometric peak force is the isometric mid-thigh pull (IMTP). Early research suggests IMTP peak force (IMTP PF) to correlate well with vertical jump variables (*r* = 0.53–0.82), such as PF, peak power, and jump height (JH) in a range of populations such as weightlifting [20], cycling [21], recreational training [22] wrestling [23], American football [24], and soccer [25] because of similarities in vertically directed force production and the subsequent biomechanically derived variables. Recent research found IMTP testing performed in a custom rack to demonstrate high between-session reliability (ICC > 0.82) for all kinetic variables (PF, rate of force development [RFD], and impulse at 100, 200, and 300 milliseconds) in eight male collegiate athletes [26]. Additionally, IMTP PF demonstrated high within-session reliability (ICC = 0.90; CV = 6.4%) when performed using a portable rig in male academy cricketers [27]. Further, reliability analysis revealed unilateral stance IMTP PF to also demonstrate high within-session reliability (ICC ≥ 0.92; CV ≤ 6.1%) [28]. However, the between-session reliability of IMTP kinetic variables during unilateral stance IMTP requires further investigation.

The countermovement jump (CMJ) has been determined to be a reliable assessment of vertical jump ability and neuromuscular performance in various sports [29]. When examining the literature, the CMJ measures have demonstrated high between-session reliability (ICC = 0.82–0.97; CV = 1.7%–6.6%) [30,31]. Further, research by Meylan et al. [32] has demonstrated within-session reliability of unilateral CMJ jump height (CMJ JH) to be considered highly acceptable (ICC = 0.97; CV = 6.7%) and in accordance with reliability data previously reported for bilateral CMJ [30,31]. However, even though practitioners can expect good reliability for unilateral CMJ JH, reliability of other kinetic and kinematic measures often assessed during unilateral CMJ require further investigation.

Therefore, the aims of this study are to establish the between-session reliability and measurement error of isokinetic dynamometry, IMTP, CMJ, and horizontal jump testing protocols in adolescent team-sport athletes. It was hypothesized that all tests would demonstrate high reliability, similar to results previously reported in adult populations. Should this be true, these values can be used to assess and monitor training-induced changes in adolescent athletes.

## 2. Materials and Methods

### 2.1. Subjects

Seventeen adolescent athletes (8 males: age 18.1 ± 2.2 years; height 175.6 ± 3.5 cm; mass 80.2 ± 3.6 kg and 9 females: age 17.9 ± 2.6 years; height 178.5 ± 4.3 cm; mass 71.5 ± 4.5 kg) all of whom participated in cricket (males) and netball (females) volunteered for the study. Athletes attended the human performance laboratory on two occasions separated by one week, at the same time of day, to determine between-session reliability of isokinetic dynamometry, IMTP, CMJ, and horizontal jump tests. All participants had regularly (≥2 × a week) performed structured strength and conditioning training in preparation for their sport, for 12–24 months. Testing took place during the final stages of preseason training, at the end of a power mesocycle, with each testing session replacing the individuals’ normal training session. The testing order was as follows: CMJ, horizontal jumps, IMTP, and isokinetic dynamometry. Before the start of testing, athletes performed a standardised warm up, as directed by the investigator. All athletes were familiar with testing procedures, having visited the laboratory for a familiarization session prior to commencing the investigation. The investigation was approved by the institutional review board, and all provided appropriate consent to participate, with consent from the parent or guardian of all players under the age of 18. The study conformed to the principles of the World Medical Association’s Declaration of Helsinki.

### 2.2. Procedures

This study used a within-subject repeated-measures research design, whereby strength- and power-related measures were each performed on two separate testing sessions, separated by seven days. The procedures documented in this study have been previously reported [13,27,28,33]. The readers are directed to these studies for a more detailed description of the test designs, equipment, and administration. Intraclass correlation coefficients, coefficient of variation, standard error of measurement, and smallest detectable difference were used to determine reliability. Athletes were required to abstain from training for 48 h before testing and asked to maintain a consistent fluid and dietary intake on each day of testing. All testing was performed at the same time of day to minimize the effect of circadian rhythms.

### 2.3. Vertical Jump

Vertical jump assessments included bilateral and unilateral CMJ. Athletes were instructed to perform a rapid eccentric phase, immediately followed by a rapid concentric phase with the intention to jump as high as possible. Countermovement jumps were performed with the hands on the hips, and countermovement depth of the eccentric phase was self-selected by the athletes to maximize CMJ height. Athletes performed three trials, with one minute of rest between trials. Unilateral CMJ testing followed bilateral CMJ testing, using the same procedures outlined for bilateral CMJ, however was only performed with one foot on the force platform, with the other limb unsupported and flexed 90° at the knee. Athletes performed three trials on each limb, with one minute of rest between trials.

Countermovement jump data were collected using a portable force platform sampling at 1000 Hz (Kistler Instrument Corporation, Winterthur, Switzerland, Model 9286AA, SN 1209740). The force platform was interfaced with a laptop to allow for direct measurement of force-time characteristics, and then analysed using Bioware software (version 5.11; Kistler Instrument Corporation, Switzerland) and applied to a customised analysis spreadsheet. Prior to the onset of the countermovement, athletes remained stationary on the force platform for one second to enable an accurate measurement of body weight. Vertical ground reaction force data were then averaged across the first second, and the onset of the countermovement was determined when this value was reduced by 5 SDs [34]. Countermovement jump movement time was calculated from the force-time record as the length of time between the onset of the countermovement and the point of take-off. Reactive strength index-modified (RSImod) was calculated by dividing the jump height by the movement time. Jump height was calculated based on the vertical displacement of the centre of mass. Additionally, jump height was calculated from flight time ([9.81 × flight time^2^]/8). Vertical velocity at take-off was obtained by dividing the resultant force–time record by body mass to give the acceleration–time record, and then numerically integrating with respect to time using the trapezoid rule. Peak force was determined from the unfiltered force–time history [35]. Peak concentric power was then taken as the product of the vertical velocity and VGRF at corresponding time points. The mean performance from each of the three trials for each condition was used for further analysis.

### 2.4. Horizontal Jump

The SBJ and SLH tests were used as measures of horizontal jump performance. Each test began with athletes placing the toes of both feet on the back of the start line, before balancing on the leg(s) to be tested. Athletes were instructed to use a countermovement, and no restrictions were placed on body angles attained during the preparatory phase. Athletes were instructed to perform the SBJ and SLH with their hands-on hips. For the SLH, athletes hopped as far forward as possible, taking off from one leg, before landing on the same leg. Three maximal trials were recorded on each leg, with one minute of rest between trials. Athletes had to land without performing a corrective step to maintain balance for the trial to be counted. If the subject did not do this, the trial was disregarded and another was attempted. The distance was measured to the nearest 0.01 m. The mean performance of the SBJ and mean score of each leg from SLH testing were used for further analysis.

### 2.5. Isometric Strength

Isometric strength was assessed during bilateral and unilateral stance isometric mid-thigh pull (IMTP) testing, using a portable force platform sampling at 600 Hz (400 Series Performance Force Plate; Fitness Technology) [27]. For the bilateral IMTP, athletes obtained self-selected knee and hip angles based on the reports of previous research [26]. For this test, an immovable, collarless steel bar was positioned at approximately mid-thigh, just below the crease of the hip, using a portable IMTP rig (Fitness Technology, Adelaide, Australia). Each athlete was provided two warm-up pulls, one at 50% and one at 75% of the athletes perceived maximum effort, separated by one minute of rest. Athletes performed three maximal IMTP, with the instruction to pull against the bar with maximal effort as quickly as possible, and push the feet down into the force plate; this instruction has been previously found to produce optimal testing results [36]. Each maximal isometric trial was performed for five seconds, and all athletes were given strong verbal encouragement during each trial. Two minutes of rest was given between the maximal effort pulls.

Following the bilateral stance IMTP, athletes were provided with 5 min of rest, Unilateral stance IMTP testing followed the same procedures outlined for bilateral IMTP testing, however was only performed with one foot on the force platform, with the other limb unsupported and flexed 90° at the knee. Athletes performed a total of six unilateral maximum effort trials (three with both the left and right limbs), in a randomized order, interspersed with two minutes of recovery between trials. The maximum force recorded from the force-time curve during the five second IMTP trial was reported as the PF for both bilateral and unilateral stance conditions, and was presented as a value relative to body mass (N∙kg^−1^). The differences between two adjacent force samples were divided by the intersample time interval (0.00167 s) to calculate the mRFD. The vertical force–time curve was integrated over 100-, 200-, and 300-millisecond windows from the onset of contraction (when the vertical force increased above a threshold of 40 N) to calculate measures of impulse [26,37]. The mean performance from each of the three trials for each condition was used for further analysis.

### 2.6. Isokinetic Strength

Peak muscle torque (N∙m) of knee extensors and flexors was measured using an isokinetic dynamometer (Biodex, Biodex Corporation, Shirley, NY, USA) during maximal concentric and eccentric isokinetic knee extension/flexion at an angular velocity of 60°∙s^−1^. A specific warm-up on the isokinetic dynamometer preceded the maximal efforts. This included three submaximal (~70%) contractions and one maximal concentric contraction of the quadriceps and hamstrings. The resistance provided by the weight of the lower leg was recorded at 30° knee flexion for gravity correction purposes. Isokinetic range was inspected to ensure that peak torque was taken within ± 1°∙s^−1^ of 60°∙s^−1^. Peak torque was obtained from five repetitions for each mode throughout an arc of 90° (full knee extension = 0°). Torque values were gravity-corrected per the manufacturer’s procedures and the highest peak flexion and extension torque of the five maximal repetitions for each mode was used for further analysis. The order of tests was concentric extensor, eccentric extensor, eccentric flexor, and concentric flexor.

### 2.7. Statistical Analysis

Data was presented as either mean ± SD. Normality of data was assessed by Shapiro–Wilk statistic, and homogeneity of variance was verified with the Levene test using SPSS software (version 17.0, SPSS Inc., Chicago, IL, USA). Reliability of the variables was examined using the intraclass correlation coefficient (ICC), coefficient of variation (CV), standard error of measurement (SEM), and smallest detectable difference (SDD). To assess the magnitude of the ICC, the threshold values were 0.1, 0.3, 0.5, 0.7, 0.9, and 1.0 for low, moderate, high, very high, nearly perfect, and perfect, respectively [5]. Coefficient of variation was calculated as (CV% = SD/mean × 100). The SEM was calculated using the formula: (SD(pooled) × (√1-ICC) [38], whereas the SDD was calculated from the formula: (1.96 × (√2)) × SEM) [39]. Paired sampled t tests were used to compare measures between sessions. Standardized differences effect sizes (ES) were calculated using Cohen’s *d* = mean session 1 − mean session 2/SD(pooled) and interpreted using the scale presented by Hopkins et al. [40]. All statistical analyses were completed using SPSS 23 (IBM, New York, NY, USA), and statistical significance for all analyses was set at *p* ≤ 0.05.

## 3. Results

A Shapiro-Wilk test of normality revealed that all data were normally distributed (*p* < 0.05). Intraclass correlation coefficients and CV demonstrated high to nearly perfect between-session reliability for all kinetic and kinematic CMJ variables across bilateral and unilateral protocols (ICC ≥ 0.64; CV < 11%) (Table 1). No significant differences (*p* > 0.05; *d* ≤ 0.24) were observed between testing sessions for bilateral and unilateral CMJ measures.

Descriptive statistics for all kinetic and kinematic variables of each CMJ are presented with ICC, CV, SEM, and SDD in Table 1. Between-sessions testing demonstrated nearly perfect reliability (ICC ≥ 0.95; CV ≤ 1.77%) for all horizontal jump measures (Table 2). No significant differences (*p* > 0.05; *d* ≤ 0.08) were observed between testing sessions for horizontal jump measures.

High to nearly perfect reliability was observed for between-session unilateral stance IMTP performances (ICC = 0.63–0.96), whereas bilateral stance IMTP measures demonstrated moderate to very high reliability (ICC = 0.44–0.86) between test sessions (Table 3). Specifically, IMTP PF demonstrated very high to nearly perfect reliability across all IMTP stances (ICC ≥ 0.86; CV < 7%). Isometric mid-thigh pull RFD demonstrated the highest CV of all IMTP kinetic measures (CV ≥ 12%). There were small, yet significant differences (*p* < 0.05; *d* = 0.32–0.37) in bilateral IMTP impulse at 100, 200, and 250 ms between testing sessions. Similarly, small, yet significant differences (*p* < 0.05; *d* = 0.21–0.25) were revealed between testing sessions in IMTP L impulse at 200 and 250 ms. No significant differences (*p* > 0.05; *d* ≤ 0.18) were found between testing sessions in IMTP R measures.

Intraclass correlation coefficients demonstrated nearly perfect reliability (ICC = 0.99; ES = 0.07–0.37, *p* > 0.05; *d* ≤ 0.37) for between-session isokinetic strength measures (Table 4). The CV for concentric and eccentric strength on the right leg (6.90%–8.20%) were lower than reported for the left leg (6.80%–12.00%).

## 4. Discussion

This study was designed to determine the between-session reliability of strength- and power-related assessments in adolescent team-sport athletes. This study showed that between-session reliability was acceptable (ICC ≥ 0.50; “strong”) for all strength- and power-related variables, excluding RFD measures during bilateral stance IMTP (ICC = 0.44; CV = 16.7%), which failed to meet acceptable reliability criteria between testing sessions (CV > 15%). Additionally, despite demonstrating high ICCS and low CV (ICC ≥ 0.76; CV ≤ 9%), impulse measures at 100, 200, and 250 ms during bilateral IMTP, and at 200 and 250 ms during IMTP L revealed small, yet significant differences between testing sessions (*p* < 0.05; *d* = 0.21–0.38). Assessment of performance variables requires high reliability to make sound conclusions about whether changes seen in response to a training intervention are meaningful or not. The findings in the current study agree with previous research in adult populations, in that isokinetic dynamometry, IMTP, CMJ, SBJ, and SLH are reliable means of assessing strength- and power-related performance in adolescent male and female athletes [11,30,31,33].

The results of the current study demonstrated that the reliability of bilateral and unilateral CMJ kinetic and kinematic variables between test sessions tended to be highly reliable (Table 1). Previous investigations that have assessed between-session CMJ reliability have reported ICC values ranging from 0.82 to 0.97 in male and female athletes [30,31]. The ICC values reported in the current study (0.64–0.99) are therefore in corroboration with previous research, highlighting the high reliability of bilateral and unilateral CMJ methods of assessing bilateral and unilateral lower-body explosive performance [30,31,32]. Although previous research [32] has reported high reliability for unilateral CMJ measures, the current study observed CMJ movement time-related measures (movement time and RSImod) to observe slightly lower ICC values and higher CV on the left leg only. This may be due to subjects being right leg dominant during unilateral CMJ and thus producing more repeatable performances across all trials. Additionally, movement time is largely affected by both eccentric and concentric phases of the CMJ, thus subjects may have alternated their countermovement strategy on their left leg to allow JH to be maintained. Furthermore, no significant differences were observed in bilateral and unilateral CMJ measures between sessions (*p* > 0.05; *d* ≤ 0.24) suggesting there was no learning effect.

Research by Munro and Herrington [33] has reported high reliability for SLH distance in male and female University students, however subjects performed hops without arm restriction and only on the dominant leg. The current study performed SLH with the hands-on hips, and on both legs, which may have influenced the findings. The study reported high reliability (ICC = 0.80; SEM < 0.08 m; SDD < 0.22 m), are similar to those reported in the current study (ICC = 0.95; SEM = 0.05 m; SDD < 0.14 m), highlighting the high reliability of SLH testing as a method to assess unilateral horizontal jump performance which may aid in the creation of accurate player profiling for performance and risk of injury. The current study found no significant differences (*p* > 0.05; *d* ≤ 0.08) in horizontal jump measures between testing sessions. Furthermore, unilateral assessments are a popular means of assessing muscle strength asymmetry. Our findings suggest that SLH testing displays a high level of test-retest reliability, and can therefore can be used to potentially assess strength asymmetries between limbs in adolescent athletes.

The results in the current study suggest that IMTP force measures are reliable across testing sessions—except for bilateral stance RFD and impulse at 100, 200, and 250 ms, and IMTP L impulse at 200 and 250 ms. The ICC values for IMTP PF and impulse are similar to those previously reported in eight collegiate athletes [26]. However, the IMTP RFD reliability measures in the current study (ICC = 0.44) are somewhat below those found in the study by Comfort et al. (ICC = 0.83) [26]. Possible explanations for this finding may be due to the level of athletes used in the current study. Haff et al. [41] demonstrated that reliability values are dependent on the method of which RFD is calculated, suggesting pre-determined time bands may be more appropriate (0–100 ms, 0–200 ms) when measuring IMTP RFD. Our findings suggest based on ICCs and CV, that unilateral stance IMTP demonstrate high between-session reliability for all IMTP measures, similar to within-session reliability values (ICC ≥ 0.92; CV ≤ 6.1%) previously reported for unilateral stance IMTP PF [27,28]. However, there were small, yet significant differences (*p* < 0.05; *d* = 0.32–0.37) in bilateral IMTP impulse at 100, 200, and 250 ms and small, yet significant differences (*p* < 0.05; *d* = 0.21–0.25) in IMTP L impulse at 200 and 250 ms between testing sessions. These findings may suggest that measures deemed reliable using ICCs are not necessarily effective for monitoring. Therefore, scientists and practitioners should consider conducting unilateral stance IMTP assessments following the same protocol implemented in this study, however RFD and impulse measures should be used with caution when assessing an athlete’s isometric force production capabilities.

Previously, Sole et al. [9] reported a high reliability (ICC ≥ 0.92) for isokinetic knee flexion and extension between test sessions in 20 healthy subjects, with very similar findings to this study (ICC = 0.99). Moreover, in the current study, there were small SEMs for all isokinetic strength measures (2.48–7.70 N·m). These values are similar to those previously observed by Sole et al. (4.74–11.20 N·m) [9]. The SEM value shows the range in which an individual’s true score is likely to lie, whereas the SDD values allow practitioners and researchers to detect whether a change in score is meaningful. The current study observed SDD values for isokinetic strength between 6.87 and 21.35 N∙m. These values are in agreement with Sole et al. [9] who found SDDs of 13.14–31.04 N∙m for peak torque during isokinetic knee flexion and extension at 60°∙s^−1^. Additionally, our findings seem to lie within the reliability values (ICC ≥ 0.92; CV ≤ 5.83%; SEM ≤ 14.22 N∙m) of previous research for isokinetic knee flexor and extensor performance of the right leg in 23 male soccer and rugby athletes [13]. Thus, it can be assumed the calculated ICCs, CVs, SEMs, and SDDs indicated acceptable between-session reliability for all isokinetic strength measures (Table 4).

Some limitations exist in the current study. Firstly, the athletes examined within this study were tested at the end of preseason. Therefore, these results may only be representative of these athletes at the specific time of testing. This study was limited by the absence of maturation data on reliability measures. Research has shown that physical capabilities develop in a nonlinear fashion because of growth and maturation, which may have affected the findings in the current study.

## 5. Conclusions

The results of this study demonstrate that commonly used strength- and power-related protocols are reliable performance measures in adolescent team-sport athletes. The findings of this study can be used by practitioners and researchers to monitor changes in strength- and power-related measures in adolescent team-sport athletes to identify improvements in performance. Specifically, when looking to identify meaningful changes in performance, scientists and practitioners should look for differences of >7% CMJ height, >8% SLH distance, >10% in peak isometric force, and >5% in isokinetic peak torques. Furthermore, performance decrements greater than the SDD could be used to monitor fatigue and preparedness for training and competition.

## Figures and Tables

**Table 1:** Between-session reliability for countermovement jump measures.

Reliability Variable	Session 1		Session 2		Change in Mean		*p*	*d*	ICC	CV	SEM	SDD
	Mean	SD	Mean	SD	Mean	SD						
**Bilateral CMJ**												
Movement Time (s)	0.85	0.13	0.82	0.16	−0.03	0.09	0.191	0.22	0.79	6.19	0.07	0.19 (22.89%)
RSImod (m∙s^−1^)	0.37	0.11	0.38	0.13	0.02	0.04	0.132	0.16	0.95	6.11	0.03	0.08 (21.05%)
Jump Height* (m)	0.31	0.05	0.32	0.05	0.00	0.02	0.431	0.04	0.94	2.63	0.01	0.03 (9.68%)
Velocity (m∙s^−1^)	2.40	0.22	2.41	0.23	0.01	0.08	0.528	0.05	0.94	1.67	0.06	0.15 (6.22%)
Jump Height** (m)	0.30	0.06	0.30	0.06	0.00	0.01	0.805	0.02	0.99	1.52	0.01	0.02 (6.67%)
Peak Force (N)	1970.33	446.35	1976.36	461.40	6.03	50.85	0.723	0.01	0.99	1.10	45.40	125.83 (6.38%)
Peak Power (W)	3783.21	882.96	3850.83	1015.91	67.63	192.55	0.407	0.07	0.98	1.96	134.68	373.32 (9.78%)
**CMJ L**												
Movement Time (s)	0.81	0.14	0.84	0.18	0.03	0.14	0.411	0.18	0.65	8.81	0.09	0.26 (31.71%)
RSImod (m∙s^−1^)	0.19	0.07	0.19	0.07	0.01	0.05	0.493	0.11	0.64	10.98	0.04	0.12 (63.16%)
Jump Height* (m)	0.17	0.04	0.17	0.05	0.00	0.10	0.508	0.02	0.97	3.37	0.01	0.02 (11.76%)
Velocity (m∙s^−1^)	1.73	0.23	1.68	0.21	−0.05	0.15	0.167	0.24	0.77	3.30	0.11	0.29 (17.06%)
Jump Height** (m)	0.15	0.03	0.15	0.04	0.01	0.13	0.120	0.11	0.93	3.48	0.01	0.03 (20.00%)
Peak Force (N)	1598.78	296.33	1610.93	306.18	12.15	27.59	0.088	0.04	0.99	1.10	30.13	83.52 (5.20%)
Peak Power (W)	2351.69	480.83	2298.42	482.22	−53.28	183.79	0.249	0.11	0.93	3.27	127.40	353.13 (15.19%)
**CMJ R**												
Movement Time (s)	0.82	0.16	0.82	0.17	0.01	0.05	0.635	0.03	0.96	3.28	0.03	0.09 (10.98%)
RSImod (m∙s^−1^)	0.19	0.06	0.19	0.06	0.00	0.01	0.707	0.01	0.97	3.33	0.01	0.03 (15.79%)
Jump Height* (m)	0.18	0.04	0.18	0.04	0.00	0.01	0.332	0.04	0.98	2.38	0.01	0.01 (5.56%)
Velocity (m∙s^−1^)	1.69	0.20	1.69	0.19	0.00	0.06	0.999	0.01	0.95	1.78	0.04	0.12 (7.10%)
Jump Height** (m)	0.15	0.03	0.15	0.03	0.00	0.01	0.782	0.01	0.94	2.95	0.01	0.02 (13.33)
Peak Force (N)	1543.30	269.94	1547.60	281.09	4.30	29.18	0.552	0.02	0.99	0.89	27.56	76.39 (4.94%)
Peak Power (W)	2282.85	439.20	2266.28	408.86	−16.57	100.11	0.505	0.04	0.97	2.32	73.50	203.73 (8.96%)

Notes: RSImod = reactive strength index-modified; Jump Height* = flight time method; Jump Height** = displacement of centre of mass method; ICC = intraclass correlation coefficient; CV = coefficient of variation; SEM = standard error of measurement; SDD = smallest detectable difference.

**Table 2:** Between-session reliability for horizontal jump measures.

Reliability Variable	Session 1		Session 2		Change in Mean		*p*	*d*	ICC	CV	SEM	SDD
	Mean	SD	Mean	SD	Mean	SD						
SBJ (m)	1.75	0.26	1.77	0.23	0.02	0.05	0.119	0.08	0.98	1.77	0.03	0.10 (5.68%)
SLH L (m)	1.56	0.22	1.57	0.23	0.01	0.07	0.494	0.06	0.95	2.34	0.05	0.14 (8.97%)
SLH R (m)	1.58	0.21	1.57	0.21	0.00	0.06	0.795	0.02	0.95	2.07	0.05	0.13 (8.23%)

Notes: SBJ = standing broad jump; SLH = single-leg hop; L = left leg; R = right leg; ICC = intraclass correlation coefficient; CV = coefficient of variation; SEM = standard error of measurement; SDD = smallest detectable difference.

**Table 3:** Between-session reliability for isometric mid-thigh pull measures.

Reliability Variable	Session 1		Session 2		Change in Mean		*p*	*d*	ICC	CV	SEM	SDD
	Mean	SD	Mean	SD	Mean	SD						
**Bilateral IMTP**												
PF (N)	2514.16	675.57	2586.96	716.19	72.94	370.02	0.309	0.10	0.86	6.81	260.59	722.33 (28.32%)
PF (N·kg^−1^)	32.84	5.66	33.79	6.37	1.06	5.18	0.412	0.16	0.86	6.81	2.26	6.26 (18.79%)
RFD (N/s)	8719.20	2384.37	9523.90	2481.64	804.76	2579.69	0.217	0.33	0.44	16.67	1822.66	5052.16 (55.39%)
I100 (N·s)	83.72	19.30	89.94	19.55	6.29	11.90	0.045	0.32	0.81	9.36	8.47	23.48 (27.04%)
I200 (N·s)	190.35	38.71	205.75	44.25	15.53	27.22	0.032	0.37	0.79	9.11	19.10	52.95 (26.74%)
I250 (N·s)	255.41	50.87	276.69	61.41	21.18	39.20	0.041	0.38	0.76	9.29	27.73	76.86 (28.89%)
I300 (N·s)	332.37	72.65	357.74	82.18	25.24	49.74	0.083	0.33	0.79	8.74	35.62	98.74 (28.62%)
**IMTP L**												
PF (N)	2143.85	501.57	2209.99	555.33	66.24	185.63	0.161	0.13	0.94	3.98	129.71	359.54 (16.52%)
PF (N·kg^−1^)	28.14	4.55	28.92	4.82	0.71	2.20	0.205	0.17	0.89	3.98	1.55	4.31 (15.11%)
RFD (N/s)	6805.86	1979.08	6950.47	2595.92	144.59	1984.14	0.868	0.06	0.63	12.30	1405.98	3897.16 (56.66%)
I100 (N·s)	84.64	19.78	88.27	22.72	3.65	7.39	0.059	0.17	0.94	6.20	5.23	14.49 (16.76%)
I200 (N·s)	189.34	44.73	201.06	49.86	11.88	18.29	0.016	0.25	0.93	6.45	12.55	34.79 (17.82%)
I250 (N·s)	252.24	62.88	265.70	67.74	13.35	5.70	0.032	0.21	0.94	6.00	16.02	44.42 (17.15%)
I300 (N·s)	321.99	81.90	338.34	89.32	16.12	35.52	0.058	0.19	0.93	5.93	22.70	62.91 (19.05%)
**IMTP R**												
PF (N)	2229.08	526.40	2306.07	614.08	76.82	152.64	0.054	0.13	0.96	3.38	114.53	317.47 (14.00%)
PF (N·kg^−1^)	29.23	4.46	30.12	5.12	0.71	1.72	0.111	0.18	0.94	3.38	1.18	3.26 (10.99%)
RFD (N/s)	7174.21	2981.68	7294.38	3074.20	120.24	2148.76	0.795	0.04	0.75	15.28	1514.34	4197.54 (58.02%)
I100 (N·s)	87.15	19.03	89.35	18.20	2.29	11.29	0.414	0.12	0.82	6.86	7.90	21.89 (24.80%)
I200 (N·s)	195.06	42.08	200.67	42.77	5.71	29.08	0.430	0.13	0.77	7.99	20.35	56.41 (28.51%)
I250 (N·s)	259.77	59.96	265.81	58.94	5.88	38.81	0.541	0.10	0.79	7.59	27.24	75.51 (28.74%)
I300 (N·s)	330.39	79.23	339.76	77.98	9.411	45.86	0.410	0.12	0.83	7.15	32.41	89.83 (26.84%)

Notes: IMTP = isometric mid-thigh pull; PF = peak force; RFD = rate of force development; I100 = impulse at 100 ms; I200 = impulse at 200 ms; I250 = impulse at 250 ms; I300 = impulse at 300 ms; L = left leg; R = right leg; ICC = intraclass correlation coefficient; CV = coefficient of variation; SEM = standard error of measurement; SDD = smallest detectable difference.

**Table 4:** Between-session reliability for isokinetic strength measures.

Reliability Variable	Session 1		Session 2		Change in Mean		*p*	*d*	ICC	CV	SEM	SDD
	Mean	SD	Mean	SD	Mean	SD						
L CON Q (N·m)	186.51	64.41	198.24	58.88	11.71	22.86	0.051	0.19	0.99	8.50	6.16	17.08 (8.88%)
L ECC Q (N·m)	220.19	72.08	229.00	56.99	8.82	46.15	0.442	0.14	0.99	12.01	6.48	17.97 (8.00%)
L CON H (N·m)	140.63	27.88	131.41	20.98	−9.24	20.26	0.079	0.37	0.99	9.00	2.48	6.87 (5.05%)
L ECC H (N·m)	158.75	34.94	156.59	29.27	−2.18	16.30	0.590	0.07	0.99	6.83	3.22	8.94 (5.67%)
R CON Q (N·m)	182.96	70.76	185.88	69.24	2.92	23.16	0.611	0.04	0.99	7.82	7.00	19.40 (10.52%)
R ECC Q (N·m)	236.61	83.08	239.88	70.53	3.28	32.05	0.679	0.04	0.99	8.23	7.70	21.35 (8.96%)
R CON H (N·m)	136.91	32.06	143.51	23.92	6.60	15.87	0.106	0.23	0.99	6.88	2.82	7.81 (5.57%)
R ECC H (N·m)	155.08	39.20	159.85	32.94	4.78	20.82	0.358	0.13	0.99	7.24	3.62	10.02 (6.36%)

Notes: CON = concentric; ECC = eccentric; Q = quadriceps; H = hamstrings; L = left leg; R = right leg; ICC = intraclass correlation coefficient; CV = coefficient of variation; SEM = standard error of measurement; SDD = smallest detectable difference.
